# Adherence to medical treatment for Wilson’s disease in children and adolescents: a cohort study from Turkey

**DOI:** 10.1186/s13023-024-03113-0

**Published:** 2024-03-07

**Authors:** Mehmet Akif Göktaş, Nadir Yalcin

**Affiliations:** 1 Division of Pediatric Gastroenterology, Medical Park Göztepe Hospital, Bahçeşehir University, İstanbul, Turkey; 2 Department of Clinical Pharmacy, Faculty of Pharmacy, Hacettepe University, Ankara, Turkey

**Keywords:** Wilson disease, Medication adherence, Quality of life, Depression, Anxiety

## Abstract

**Background:**

This study aimed to assess medication adherence and demographic, clinical, and psychopathological parameters such as quality of life, depression, and anxiety levels that can affect pediatrics with Wilson’s Disease (WD).

**Methods:**

A prospective cohort study was conducted at an outpatient clinic in Turkey among pediatric patients (2 to 18 years) with WD between November 2022 and April 2023. The Medication Adherence Report Scale (MARS-5) as a subjective and Medication Possession Ratio (MPR) as an objective assessment were scored. Physical, genetic and biochemical parameters, the Pediatric Quality of Life Inventory (PedsQL) for both parents and patients, Childhood Depression Inventory, State Trait Anxiety Inventory were also administered.

**Results:**

A total of 30 pediatric outpatients who were prescribed D-penicillamine (*n* = 27) or trientine (*n* = 3) as chelators and zinc (*n* = 29) and pyridoxine (*n* = 19) as supplements were included. Proteinuria (*n* = 3), skin rash (*n* = 2), and gastrointestinal upset (*n* = 2) were observed. When the correlation between MARS-5 and duration of follow-up was examined, a significant negative correlation was found (*p* = 0.014). According to MPRs, non-adherence rates (missed doses ≥ 20%) were 29.6%, 17.2% and 5.3% for D-penicillamine, zinc and pyridoxine, respectively. PedsQL scores were higher than those of parents, with a positive correlation between them (*p* < 0.001). Also, there was a significant positive correlation between PedsQL and State Anxiety Inventory (*p* < 0.001). Comparing the change in urinary copper levels between different levels of treatment knowledge, significant differences were observed between high- and low levels (*p* = 0.043).

**Conclusions:**

Overall, nonadherence rates were 23.3% based on MARS-5 and 5.3–29.6% based on MPR. It is essential to consider factors such as the duration of follow-up, biochemical parameters, treatment knowledge, quality of life and anxiety as potential influencers of medication adherence.

## Background

Wilson’s disease (WD) is an autosomal recessively inherited disorder of copper utilization in hepatocytes caused by mutations in the ATP7B gene, with a reported prevalence of 1:30,000–50,000 [1]. Defective utilization of copper leads to hepatic copper retention and chronic liver damage. Consequently, copper toxicity can damage other organs or tissues (especially the brain, eyes, erythrocytes, kidneys and cartilage). Early diagnosis is most likely to result in near-normal longevity with good health, as long as the patient tolerates effective medication, adheres to the lifelong treatment regimen, and has consistent access to the medication [2].

Pharmacotherapy of pediatric liver diseases has made significant progress in recent decades, leading to increased survival. Effective pharmacological agents are available for common disorders such as WD, autoimmune liver disease, and some rare metabolic disorders that can avoid the need for liver transplantation (LT) in a significant number of patients [3]. On the other hand, psychiatric disorders are commonly confirmed in WD and contribute to lower quality of life, psychosocial outcomes, and medication adherence [4].

The literature includes studies that define the frequency of medication non-adherence within the 4-week recall medication adherence measure administered by the interviewer in patients with WD and other liver diseases, as well as studies that determine the barriers to adherence with the Child and Adolescent Medication Adherence Questionnaire [5, 6].

The use of zinc in WD is more relevant in young genetically verified cases with no clinical symptoms and in those with neurological disease who worsen with chelators. Its usage in conjunction with chelators has not been proved to improve efficacy, increases the number of drugs, and frequently results in an undetectable and thus suboptimal regimen due to the need to separate the administration timing of chelators, zinc, and food intake. Zinc should only be used in such circumstances if there is a clear deficiency [7].

The details of children with WD’s cognitive symptoms that affect adherence are not obvious from the current literature. Therefore, we aimed to determine medication adherence with objective/subjective measurements and demographic and clinical parameters that affect medication adherence in pediatric patients with WD.

## Methods

### Study participants

In this prospective cohort study, children aged 2–18 years who were being at least 6 months followed up in the Department of Pediatric Gastroenterology of Hacettepe University İhsan Doğramacı Children’s Hospitals with the diagnosis of WD were included in the study during the 6-month data collection period (between November 2022 and April 2023).

#### Inclusion criteria

- Diagnosis of WD by clinical, laboratory, liver histopathology, genetic methods and exclusion of other possible differential diagnoses.
- Obtaining consent for the study from the patients (> 12 age) and parents/legal guardians.

#### Exclusion criteria

- Family or patient unwillingness to participate in the study.
- Newly diagnosed patients with less than 6 months of follow-up period.

#### Data acquisition

The medications they were taking and their doses, medication adherence, physical examination findings were recorded on the patient follow-up forms. Pediatric gastroenterologists, who deal with patients individually, subjectively examined their patients’ level of treatment knowledge and their perception of the illness, categorized as low, medium or high. Full blood count, aminotransaminase levels, and 24-hour urine copper results were noted on the same follow-up forms. Patients’ socioeconomic and sociocultural levels (SSL), quality of life, depression and anxiety levels, and medication adherence were determined by some scales as described below. According to the Hollingshead-Redlich Scale, socioeconomic and sociocultural levels (SSL) of the patients were determined. The highest SSL was classified as Class 1 and the lowest as Class 5 [8]. In our study, the numbers in each class were regrouped at three levels (high level: Class 1 and 2, medium level: Class 3, low level: Class 4 and 5). The Pediatric Quality of Life Inventory (PedsQL; completed by parents and patients aged 5 to 18) which higher scores indicate better health-related quality of life, Childhood Depression Inventory (completed by only patients aged 7 to 18) and State Trait Anxiety Inventory for Children and Parents (completed by only patients aged 5 to 18) which higher scores indicate higher depression and anxiety levels were applied to the patients. For Childhood Depression Inventory (range: 0–54), the cut-off scores were determined in order of severity of depression: 15 for mild, 20 for moderate, and 25 for severe depression with high sensitivity and specificity [9]. For State Trait Anxiety Inventory scores range from 20 (low anxiety) to 80 (high anxiety) [10]. A cut-off score of 40 is commonly used to define probable clinical levels of anxiety [11]. In addition, the Turkish version of the Medication Adherence Report Scale (MARS-5) as a subjective assessment and the Medication Possession Ratio (MPR) as an objective assessment were scored for only patients to determine medication adherence [12]. It was predicted that higher MARS-5 scores would be associated with stronger necessity beliefs and lower MARS-5 scores would be associated with stronger concerns (non-adherence cut-off score: <20). Also, MPR, which shows the ratio of the number of days a patient is kept in stock for their medication, to the number of days a patient should keep in stock for their medication, takes a value between 0 and 1 and a value close to 1 indicates high adherence rates. In line with this ratio, non-adherence was defined as the percentage of dose missed ≥ 20%.

According to the National Pediatric WD Treatment Guideline, the maintenance dose for D-penicillamine was 10–15 mg/kg/day and 20 mg/kg/day for triethylenetetramine (trientine). Elemental zinc was administered as 150 mg/day divided into three doses if older than 16 years and body weight > 50 kg, 75 mg/day divided into three doses if 6–16 years and body weight < 50 kg, and 50 mg/day divided into two doses for those younger than 6 years. Pyridoxine (vitamin B*6*) was added routinely to the treatment regimen in a dosage of 20–50 mg daily [13].

The study protocol was approved by the local Ethics Committee. The patients and the parents who met the inclusion criteria for the study were informed about the study, and informed consents were obtained from participants or parents/legal guardians.

### Statistical analysis

Firstly, it was planned at the beginning of the study to include at least 26 patients with an effect size of 0.60, a power of 95%, and a margin of error of 5% (G* Power 3.1 Statistical Power Analysis) based on the literature [14]. The normality of continuous variables was tested using the Shapiro–Wilk test. After data extraction, continuous variables were defined as the mean ± standard deviation (SD) and median (range), depending on the result of normality test. Wilcoxon, Mann–Whitney U tests and post-hoc analysis was used to compare quantitative data, McNemar test to compare qualitative data, and Spearman correlation coefficient to investigate the correlation between variables. For all tests, *p* < 0.05 was considered statistically significant. All analyses were carried out in the *IBM SPSS Statistics Version 23* software.

## Results

### Demographic and clinical profile

A total of 30 pediatric outpatients with WD, mostly with liver involvement only (*n* = 29), were included in the study. Of these, 15 (50%) patients were diagnosed with elevated liver transaminase levels. The diagnosis of WD was genetically confirmed in 24 patients and clinically confirmed in 6 patients. While most patients (73.3%) had a family history of WD, the rate of consanguineous marriages, which is common in Turkey, was 56.7%. The rates of patients with low disease knowledge, treatment knowledge and illness perception were 33.3%, 36.7% and 46.7%, respectively. Twenty-seven (90%) patients were treated with D-penicillamine and the remaining 3 (10%) patients were treated with trientine. Zinc and pyridoxine supplements were taken by 96.7% and 63.3% of patients, respectively.

During the study period, D-penicillamine-related proteinuria occurred in three patients, skin rash in two patients, and zinc-related gastrointestinal symptoms (vomiting, upset stomach) occurred in two patients (Table 1).

**Table 1:** Demographical features (*N* = 30)

Variables	n (%)
Gender, female	17 (56.7)
Age (year), mean (SD)	13.75 (3.22)
z-score for weight (kg), median (range)	−0.02 [−1.26−(+ 3.58)]
z-score for height (cm), median (range)	0.08 [−1.86−(+ 1.53)]
z-score for body mass index (kg/m^2^), median (range)	0.21 [−3.20−(+ 2.59)]
Age of onset of Wilson’s disease (year), mean (SD)	7.04 (3.51)
Duration of follow-up of Wilson’s disease (year), mean (SD)	6.74 (4.26)
**Symptoms/findings that require investigation for diagnosis**	
Elevated liver transaminase levels	15 (50.0)
Sibling screenings	10 (33.3)
Jaundice	3 (10.0)
Focal fatty liver on ultrasonographic examination	1 (3.3)
Extrapyramidal symptoms	1 (3.3)
**İnvolved organs**	
Liver	29 (96.7)
Brain	1 (3.3)
**Caregiver**	
Parents	28 (93.3)
Grandparents	2 (6.7)
**Education**	
Elementary school	5 (16.7)
Middle school	11 (36.7)
High school	13 (43.3)
Bachelor	1 (3.3)
**Socioeconomic status**	
Low	8 (26.7)
Medium	17 (56.7)
High	5 (16.7)
Family history of Wilson’s disease	22 (73.3)
Consanguineous marriage	17 (56.7)
**Disease knowledge**	
Low	10 (33.3)
Medium	4 (13.3)
High	16 (53.4)
**Treatment knowledge**	
Low	11 (36.7)
Medium	3 (10.0)
High	16 (53.3)
**Illness perception**	
Low	14 (46.7)
Medium	1 (3.3)
High	15 (50.0)
**Medications**	
D-penicillamine	27 (90.0)
Trientine	3 (10.0)
Zinc	29 (96.7)
Pyridoxine (vitamin B_6_)	19 (63.3)
Other medications (vitamin D, E, iron, enalapril, sertraline, lansoprazole, etc.)	8 (26.7)
**Adverse effects**	
D-penicillamine related proteinuria	3 (10.0)
D-penicillamine related skin rash	2 (6.7)
Zinc related gastrointestinal symptoms (vomiting, stomach upset)	2 (6.7)
Kayser-Fleischer rings	3 (10.0)
ATP7B genotype	24 (80.0)
Ceruloplasmin, mg/dl (onset of treatment), median (range) (*n* = 30)*	5.4 (2.0–44.6)
Hepatic copper, µg/g dry weight (onset of treatment), median (range) (*n* = 16)**	502.0 (4.5–1983.0)
Urinary copper excretion, µg/24 h (onset of treatment), median (range) (*n* = 10)***	497.5 (107.0–1075.0)

*Reference level: <20 mg/dl

**Reference level: >250 µg/g

***Reference level: >100 µg/24 h

### Measures of adherence, quality of life and psychopathology

The fact that the mean (SD) MARS-5 score [20.67 (4.55)], which is the subjective indicator of medication adherence, was close to 25 indicates that the patients have good adherence to WD treatment. When the correlation between MARS-5 and follow-up time was examined, a significant negative correlation was found (*r*=−0.466, *p* = 0.014). In addition, according to the objective adherence indicator MPR, the rate of adherence to primary pharmacotherapy (D-penicillamine and trientine) and supplements (zinc and pyridoxine) was high (0.89 to 1.00) (Table 3). According to the MPRs, non-adherence rates (missed doses ≥ 20%) were 29.6%, 17.2% and 5.3% for D-penicillamine, zinc and pyridoxine, respectively.

When the adherence rates of patients using D-penicillamine or trientine were compared according to the MARS and MPR, no significant difference was found (mean rank: 15.65 vs. 14.17 for MARS-5 and 15.48 vs. 15.67 for MPR, *p* > 0.05). When comparing the subjective (MARS-5) and objective (MPR) zinc adherence rates of patients using D-penicillamine or trientine, no significant difference was found (mean rank: 15.81 vs. 8.00 for MARS-5 and 15.31 vs. 12.33 for MPR, *p* > 0.05).

When the correlation between MPR for D-penicillamine and PedsQL was examined, a significant negative correlation was found (*r*=−0.423, *p* = 0.028). There was also a strong positive correlation between objective (MPR) and subjective (MARS-5) indicators of medication adherence (*r* = 0.693, *p* < 0.001). According to the cut-off values for both scales, there was also a significant correlation between them (Cramer’s V: 0.558, *p* = 0.007). On the other hand, the patients’ PedsQL (21.60) was higher than the parents’ (19.30). When the correlation between the two scores was examined, a significant positive correlation was found (*r* = 0.747, *p* < 0.001). There was also a significant positive correlation between the PedsQL and the State Anxiety Inventory for Children (*r* = 0.638, *p* < 0.001).

According to the liver transaminase and International Normalized Ratio (INR) levels, a statistically significant improvement was observed in patients compared to the start of the medical treatment (*p* < 0.05) (Table 2).

**Table 2:** Comparison of laboratory values at the onset of treatment and at present

Parameters	Onset of treatment	At present	P value
ALT, UI/L, median (range)	164.5 (26–1778)	45.5 (12–299)	**< 0.001**
AST, UI/L, median (range)	87 (26–348)	37.5 (12–199)	**< 0.001**
GGT, UI/L, median (range)	50 (14–277)	26 (11–92)	**0.007**
ALP, UI/L, median (range)	285.5 (74–488)	237.5 (44–439)	**0.014**
Total bilirubin, mg/dL, median (range)	0.46 (0.19–5.80)	0.65 (0.26–1.84)	**0.048**
Direct bilirubin, mg/dL, median (range)	0.85 (0.01–2.04)	0.14 (0.06–0.98)	0.157
Albumin, g/dL, median (range)	4.43 (1.22–5.03)	4.38 (3.76–5.40)	0.510
INR, median (range)	1.14 (0.97–4.77)	1.01 (0.92–1.22)	**< 0.001**
Urinary copper, µg/24 h, median (range)	113.0 (5.0-618.0)	288.6 (0.97-1793.37)	**0.018**

ALT: Alanine transaminase, AST: Aspartate aminotransferase, GGT: Gamma-glutamyl transferase, ALP: Alkaline phosphatase, INR: International normalized ratio, Boldface font indicates statistically significant variable (*p* < 0.05)

According to the cut-off scores (< 20 points), the mean Childhood Depression Inventory score (7.07) indicates that the mild depression symptoms for the study population. Also, according to the cut-off scores (< 40 points), mean State Trait Anxiety Inventory scores (33.30 and 36.97, respectively) indicate that low anxiety levels for the study population (Table 3).

**Table 3:** Adherence, quality of life and psychopathology measurements of the patients

Scales	Onset of treatment
**MARS-5, mean (SD)**	20.67 (4.55)
Non-adherence at a cut-off of < *20, n (%)*	7 (23.3)
**MPR, median (range)**	
D-penicillamine (*n* = 27)	0.96 (0–1.00)
Non-adherence at a cut-off of ≥ 20%, n (%)	8 (29.6)
Trientine (*n* = 3)	0.89 (0.83–0.99)
**Non-adherence at a cut-off of ≥ 20%, n (%)**	–
Zinc (*n* = 29)	0.93 (0–1.00)
Non-adherence at a cut-off of ≥ 20%, n (%)	5 (17.2)
Pyridoxine (vitamin B_6_) (*n* = 19)	1.00 (0.03–1.00)
Non-adherence at a cut-off of ≥ 20%, n (%)	1 (5.3)
PedsQL for Children, mean (SD)	21.60 (13.40)
PedsQL for Parents, mean (SD)	19.30 (11.78)
Childhood Depression Inventory, mean (SD)	7.07 (5.81)
**State trait anxiety inventory for children, mean (SD)**	
State Anxiety	33.30 (10.55)
Trait Anxiety	36.97 (11.17)

MARS-5: The Medication Adherence Report Scale, SD: Standard deviation, MPR: Medication Possession Ratio, PedsQL: The Pediatric Quality of Life Inventory

When the change in urinary copper levels from the start of treatment to the present was compared between treatment knowledge levels using post-hoc analysis, significant difference was found between high- and low-treatment knowledge levels (mean difference: 416.49, *p* = 0.043).

## Discussion

The current study found that good adherence to medication and supplements according to both objective and subjective tools, and there was also a strong positive correlation between the two. However, there was no significant difference in adherence between D-penicillamine and trientine. When we assessed quality of life and psychopathology for each patient, quality of life was higher in children than in their parents, and there was a positive correlation between them.

It is well known that medication adherence is relatively poor in asymptomatic chronic diseases [7]. On the other hand, poor adherence is associated with the development of some neurological impairments in patients with WD. For this reason, future clinical care of pediatric WD, including pre-symptomatic patients, should include proactive methods to promote and maintain adherence to the prescribed pharmacotherapy plan [2]. Highlighting the importance of medication adherence, a study by Suchismita et al. compared non-adherence between paediatric patients with WD and autoimmune liver disease and post-LT recipients and found that the WD group had significantly higher non-adherence compared to the post-LT group in terms of missed doses (20% vs. 0%) and late doses (16.7% vs. 2.4%) at a cut-off of ≥ 20% (*p* = 0.016). In our study, late dose was not calculated. However, according to the MPR, which shows the rate of missed doses within the last month, the non-adherence rate in our population was 29.6%, higher than in Suchismita’s study [15].

Its use in WD is controversial because the use of zinc in combination with chelators does not improve efficacy, increases polypharmacy and often leads to an uncontrolled and therefore sub-optimal regimen, as chelators, products containing polyvalent cations (calcium, zinc, iron, etc.) and food intake should be separated due to the formation of nonabsorbable chelates [3, 14]. However, in our study, 82.8% of the patients showed good adherence to zinc and strictly followed the instructions that penicillamine and zinc co-administration should be separated by at least 1 h due to pharmacokinetic interaction at the absorption level, thus ensuring optimal chelator therapy in the target serum zinc range.

In a retrospective study, treatment was effective in 81% of 26 patients (D-penicillamine = 13, trientine = 8, and zinc = 5) who received a single daily dose of chelators, with no safety concerns [16]. In another prospective study, all patients (*n* = 8) remained clinically well on once-daily trientine [17]. However, in our study, medication adherence in 30 patients was slightly higher according to the median MPR (96% for D-penicillamine and 89% for trientine), despite the fact that all patients received two daily doses of chelators. When the safety profile of the chelators was evaluated, D-penicillamine-related skin rash was observed in only two patients. To summarize, switching to a single daily dose may help to improve long-term treatment adherence and persistence in pediatric patients with WD. However, similar to our study, there is a need for larger, prospective studies that include both treatment safety/efficacy and patient quality of life in the long-term maintenance phase.

In a retrospective analysis, unlike our study, 74.1% of WD patients were irregular in their anti-copper treatment, which had a significant negative impact on the clinical response [18]. However, the low adherence rate of 32.4% in another cross-sectional study shows that adherence may vary between societies due to variability in health policy, symptom severity, treatment protocol, mutation, and primary copper accumulation [19]. According to another study evaluating treatment persistence (defined as the length of time for which the patient takes the prescribed medication at the required dose) in patients with WD, significantly better self-assessment results were found (deterioration: none in the persistent group vs. 42.3% in the non-persistent group, *p* < 0.0001). On the other hand, the visual analogue scale (VAS) assessment of well-being was better in favor of the persistent patients (75.93 vs. 77.04, *p* = 0.056) [20]. In our study, both objective and subjective adherence were assessed and found to be positively correlated with each other as added value. In addition, a more comprehensive quality of life scale for both parents and children were used instead of the VAS, a simple tool to measure well-being.

In a retrospective study evaluating the efficacy of and adherence to trientine and/or zinc in children with WD (*n* = 22), non-adherence is a common cause of elevated aminotransaminase levels [21]. Differently, in our study, we found no correlation between all transaminase changes and MARS/BARS.

According to a study evaluating patients over 14 years of age including adults with WD, levels of exchangeable (free) copper were significantly lower in patients with high or medium adherence compared to low adherence (0.67 vs. 0.80 µmol/L; *p* = 0.049) [19]. In our study, which is guiding only for childhood, a significant difference was found between high and low treatment knowledge levels in terms of change in urinary copper levels (*p* = 0.043).

Since only pediatric patients were studied in a population with a rare disease, the sample size was limited. The study was conducted in a single center, but it was not considered a disadvantage because it was conducted in the largest children’s hospital center where patients were evaluated nationally. On the other hand, due to the neuropsychiatric complications of the disease and the fact that they were in the period of child development, it was a challenging process to scales that take a long time to apply to the pediatric patients and to focus their attention.

## Conclusion

It was found that almost half of the patients included in the study did not have a high level of disease and treatment knowledge and disease perception. Objectively and subjectively assessed medication adherence were determined to be related to each other, with almost a quarter of the patients being non-adherent, experiencing mild adverse effects that do not interfere with treatment. Also, it was found that the quality of life of the parents was lower but correlated with their children. The results of our study need to be supported by larger prospective randomized controlled trials with long-term evaluation of biochemical analyses and neuropsychiatric scales.

## Data Availability

The data presented in this study are available on request from the corresponding author. The data are not publicly available due to restrictions privacy and ethical.
